# Expression of barrier proteins in the skin lesions and inflammatory cytokines in peripheral blood mononuclear cells of atopic dogs

**DOI:** 10.1038/s41598-021-90992-z

**Published:** 2021-06-01

**Authors:** Sarita Kanwal, Shanker K. Singh, Sandeep P. Soman, Soumen Choudhury, Priyambada Kumari, Pradeep K. Ram, Satish K. Garg

**Affiliations:** 1 Department of Veterinary Medicine, College of Veterinary Science and Animal Husbandry, Uttar Pradesh Pandit Deen Dayal Upadhyaya Pashu-Chikitsa Vigyan Vishwavidyalaya Evam Go Anusandhan Sanstahan (DUVASU), Mathura, U.P. 281 001 India; 2 Department of Veterinary Pharmacology and Toxicology, College of Veterinary Science and Animal Husbandry, Uttar Pradesh Pandit Deen Dayal Upadhyaya Pashu-Chikitsa Vigyan Vishwavidyalaya Evam Go Anusandhan Sanstahan (DUVASU), Mathura, U.P. 281 001 India; 3 College of Biotechnology, Uttar Pradesh Pandit Deen Dayal Upadhyaya Pashu-Chikitsa Vigyan Vishwavidyalaya Evam Go Anusandhan Sanstahan (DUVASU), Mathura, U.P. 281 001 India

**Keywords:** Immunology, Molecular medicine, Pathogenesis

## Abstract

Atopic dermatitis (AD) is one of the most common skin diseases of dogs. Defects in the skin barrier and overproduction of inflammatory cytokines may be the pathogenesis of canine AD. Therefore, the present study was aimed to quantify the gene expression of certain skin barrier proteins and inflammatory cytokines in dogs with AD. Eleven dogs with AD and three healthy dogs were included in the present study. The skin barrier proteins, namely *Filaggrin* (*FLG*) and *Involucrin* (*IVL*), gene expression was quantified by Real-time PCR in the lesional skin tissues of the atopic dogs and normal skin of the healthy dogs. In addition to the skin proteins, the gene expressions of the interleukin (IL)-13, IL-31, and tumour necrosis factor (TNF)-α were also quantified in the peripheral blood mononuclear cells (PBMCs) of these dogs. Compared to the healthy dogs, significantly higher (*P* ≤ 0.01) *FLG* gene expression and significantly (*P* ≤ 0.05) lower expression of the *IVL* gene were quantified in the skin of atopic dogs. Further, the dogs with AD revealed significantly higher expression of TNF-α (*P* ≤ 0.01), IL-31 (*P* ≤ 0.05), and IL-13 (*P* ≤ 0.05) as compared to the healthy dogs. The findings of our present study evidently suggest significantly increased and decreased expressions of *FLG* and *IVL* genes, respectively, which may be responsible for disruption of the skin barrier in dogs with AD. While, the over-expressions of TNF-α, IL-31, and IL-13 genes might be attributed to the clinical pathology and manifestations of AD in dogs. However, further studies are warranted to substantiate our hypothesis about pathogenesis and clinical manifestation of AD in dogs by including a large number of animals.

## Introduction

Atopic dermatitis (AD) is a common chronic inflammatory skin disease of dogs which shares 3 to 58 per cent of canine skin diseases presented to veterinarians^1^. A combination of genetic, environmental and immunological factors seems to be responsible for AD and influence its pathogenesis and outcome. The epidermal structural, functional and compositional changes of dogs may predispose to the development of AD by enhancing percutaneous absorption and antigen presentation to the immune system^2,3^. The enhanced penetration of the environmental allergens owing to defective skin barrier is attributed to activation of the pro-inflammatory cytokines in dogs with AD^4–6^. The cornified envelop (CE) proteins such as *Filaggrin* (*FLG*) and *Involucrin* (*IVL*) in the stratum corneum play crucial role in the permeability barrier of the skin. Keratinocytes proliferation and differentiation results in synthesis of several CE proteins including *FLG*, loricrin, *IVL*, and S100 in the granular layer^7,8^. In the final epidermal differentiation, CE proteins help to form the outermost cornified layer of the epidermis and hydration of the skin. *Filaggrin* has been shown to be a key player in the pathogenesis of AD in human patients and dogs^9,10^. The epidermal barrier proteins expression, *FLG* and *IVL*, in dogs has been investigated by some researchers, however, their precise role in the development of canine atopic dermatitis is yet not well elucidated^2,11,12^.

Apart from the role of the skin barrier function, immunological hyper-reactivity has also been strongly suggested to be associated with the pathogenesis of atopic dermatitis in humans and dogs. The regulation and direction of the nature of immune responses are commonly attributed to various cytokines^13,14^. Allergic diseases of humans and dogs could be the result of an inappropriate production of type 2 immune cytokines. The production of IL-4 and the homologous cytokine IL-13 are paramount drivers of type 2 immune response^14^. Another type 2-derived cytokine is IL-31 which is predominantly produced by the Th2 lymphocytes infiltrating the skin in AD^5,15^. Interleukin-31 has been recently identified as a potent pruritogenic cytokine which induces itch in AD and other pruritic inflammatory skin disorders in humans and dogs^5,16–18^. Interferon (IFN)-γ is the main Th1 cytokine; however, TNF-α and IL-2 are also secreted from Th1 cells. The pro-inflammatory cytokines are considered to have a key role in the development of itch sensation and inflammation, which are the hallmark of AD. Moreover, this inflammation down regulates the expression of barrier proteins and exaggerates epidermal barrier dysfunctions in human patients^19,20^. To date, no detailed studies seem to have been undertaken with an insight into the alterations in the skin barrier proteins expression, and their association with the pruritogenic cytokines expression in dogs with AD. Therefore, the present study aimed to investigate the expression of the skin barrier proteins (*FLG* and *IVL*) and alterations in levels of inflammatory cytokines (TNF-α, IL-31, and IL-13) to establish an association between the skin barrier proteins and inflammatory cytokines expressions in dogs with AD.

## Materials and methods

### Animals

Total 11 clients-owned dogs suffering from dermatological disorder (AD) and presented for the treatment in the Teaching Veterinary Clinics (Kothari hospital) of College of Veterinary Science and Animal Husbandry, DUVASU, Mathura, were included in the present study. Of the 11 atopic dogs, seven (63.6%) were intact males and remaining four were intact females. Out of these 11 AD dogs, five were Labrador Retrievers, two each of Dogo Argentino and Pugs*,* and one each of the German Shepherd and Beagle breed. The age of the diseased dogs ranged from 2 to 8 years (mean 4.5 years). Three healthy clients-owned dogs (two Labrador retrievers and one Germen shepherd) which were brought to our clinics for routine health check-up and vaccination were included in the present study and kept as controls. Of these three health dogs, two were intact males and one was intact female. They were between 2 and 5 years-age.

The diagnosis of canine AD was based on the history, clinical signs and compliance to the Favrot’s criteria^21^. The dogs which fulfilled any five of the Favrot’s AD inclusion criteria i.e. (1) revealed onset of signs under three years of age, (2) living mostly indoors, (3) had glucocorticoid-responsive pruritus, (4) had pruritus without lesions at onset, (5) had affected front feet with clinical lesions (6), had affected ear pinnae, (7) had non-affected ear margins and (8) had non-affected dorso-lumbar area were included in the present study. All these dogs were also ardently examined for flea infestations and flea excrement by using flea combs. The diseased dogs that were free from flea infestation and did not have flea excrement were only included in the present study. Furthermore, to detect the mange mites infestation, skin scarping examination was performed. The diseased dogs detected free from any mange mites infestations and who had not revealed any pinnal-pedal scratch reflex on clinical examination were only included in the present study. Further, the dogs included in the present study also had the history of chronic dermatitis and were non-responsive to acaricides. Dogs were also ascertained negative for any of the haemoprotozoan diseases based on thin blood smear examination and had the physiological parameters like body temperature, respiratory-rate and heart-rate within the normal reference range. It was also ascertained that none of the dog included in this study had been treated with glucocorticoids, cyclosporin, oclacitinib, lokivetmab, antihistamines, essential fatty acids or ear products containing glucocorticoids during the last 30 days prior to presentation in our veterinary clinic for treatment. None of the enrolled dogs were receiving allergen-specific immunotherapy. Diagnosis of the concurrent pyoderma and yeast infestation was also made by cytologic examination of the pressure slide impression smears which were prepared from the skin lesions of each participated dog. Bacterial and yeast infections were controlled before inclusion.

Further, clinical evaluation of the canine atopic dermatitis lesion index (CADLI)^22^ and pruritus visual analogue scale (pVAS) based on owner-derived data^23^ were also assessed for each of the atopic dogs. Briefly, CADLI was considered only onto the body regions most frequently affected in atopic dogs (head and pinnae, forefeet, hind feet, ventral thorax and axillae, ventral abdomen and inguinal region). The scores of two lesions type subclusters as “erythema-excoriation-erosion” which was referred to as CADLI1 and as “alopecia-lichenification-hyperpigmentation” which was referred to as CADLI2 on a six-point ordinal scale were recorded onto each of the indicated body regions. The integrated severity and extent of the lesion(s) in the area were scored as 0 = none; 1 = mild; 2, 3 = moderate; 4, 5 = severe and extensive lesions (total CADLI between 0 and 50). To evaluate the level of pruritus (pVAS), the pet owners were asked to indicate on a 10-cm-long vertical line where their dog’s pruritus could be placed. The length (in mm) from top to the owner’s sign was measured and used as the tool for quantifying the animal’s pruritus (score ranging from 0 to 10).

### Isolation of RNA from the blood and skin biopsy samples

With the written informed consent of the pet owners, 4 mm skin biopsy samples of the lesional skin of dogs with AD, and from normal skin of the healthy dogs were collated with the help of sterilised skin punch biopsy. Before obtaining the punch biopsy, the selected skin lesion was locally anaesthetized by topical application of local anaesthetic, 2% lignocaine jelly. Further, the biopsied skin samples were immediately transferred to liquid nitrogen to prevent the degradation of the RNA. Blood samples (6 ml) were collected from each of the dogs with AD into heparinized tubes and used for isolation of peripheral blood mononuclear cells (PBMC). Blood samples were also collected from the healthy control group in a similar fashion. Total RNA was extracted from the skin tissues by using the PureLink® RNA mini kit. The RNA from blood was extracted by the manual method by using TRIZOL reagent and a phenol/chloroform/isopropyl alcohol method. Isolated RNA was checked for its purity and concentration by using Eppendorf Biophotometer plus. Absorbance was recorded at 260 nm and 280 nm against nuclease-free water as blanks. RNA samples showing A 260/280 value 1.8 in the PBMCs and ratio 1.9–2.0 in the skin samples were used. RNA with a concentration up to 1000 ng was used for cDNA synthesis.

### Complementary DNA (cDNA) synthesis

Complementary DNA (cDNA) was synthesised from the mRNA present in the total RNA using Revertaid® First-strand cDNA synthesis kit (Thermo Scientific, USA), following the manufacturer's instruction in thermal cycler using 1000 ng of total RNA. The reaction was carried out in 20 µl of the reaction mixture. 1000 ng of RNA was used for each reaction and the final volume was adjusted by adding nuclease-free water.

### Polymerase chain reaction (PCR)

The cDNA was checked for quality by performing PCR with standard GAPDH and RPS 19 primers. The polymerase chain reaction was standardized for each gene using synthesised cDNA. The reaction was carried out at different annealing temperatures and the optimum annealing temperatures for different genes were determined. The protocol suggested by Sambrook and Russel^24^ was followed. Endpoint optimization was done by using DreamTaq PCR master mix (Thermo Scientific). Primers used for specific genes were suggested by Theerawatanasirikul et al.^25^ (Table 1).

**Table 1:** Gene sequences of the skin barrier proteins and cytokines primers.

Genes	Primers length (kb)		Gene sequence
*Involucrin* (*IVL*)	158	F R	AAAGAAGAGCAGGTGCTGGA TGCTCACTGGTGTTCTGGAG
*Filaggrin (FLG)*	203	F R	GATGACCCAGACACTGCTGA TGGTTTTGCTCTGATGCTTG
TNF-α	121	F R	AGCCAGTAGCTCATGTTGTAGCAA GGCACTATCAGCTGGTTGTCTGT
IL-13	71	F R	GCGGCAGGGCAGATTTC AGGTTTTTCACCAACTGGATCACT
IL-31	188	F R	CCTGTTCCTGCTCTGCTCTA TGAGACACAGCAGCAAGGTA
RPS 19	98	F R	CCTTCCTCAAAAA/GTCTGGG GTTCTCATCGTAGGGAGCAAG
GAPDH	98	F R	AAGGCTGAGAACGGGAAACT TACTCAGCACCAGCATCACC

### Quantitative real-time RT-PCR reaction

Real-time RT-PCR was performed using SYBR Green master mix (PowerUpTMSYBRTM Green master mix [2X]; Thermo Fischer Scientific, USA). Each sample was run in duplicate in 10 µl reaction. The reaction mixture of 10 µl consisted of 5 µl SYBR Green master mix, 0.5 µl from 10 pmol/µl stock solution of each of the gene-specific forward and reverse primers, and 1.0 µl of cDNA (1:3 dilution) and volume was adjusted to 10 µl with nuclease-free water (NFW). To assess the specificity of amplified product, dissociation curve was generated. The results are expressed as threshold cycle values (CT). The melt curve plots and amplification plots for *FLG*, *IVL* and *RPS19* in the skin punch biopsy samples, and TNF-α, IL-31, IL-13 and *GAPDH* in PBMCs were recorded for all of the participated dogs.

To study the relative change in gene expression, the 2^−ΔΔC^_T_ method was used as described by Livak and Schmittgen^26^. The formula used to calculate the “fold change in mRNA expression” was = 2^−ΔΔC^_T_,” [where ΔΔC_T_ = (C_T,target gene_ − C_T,reference gene_) treatment − (C_T,target gene_ − C_T, reference gene_) control]. The gene-specific amplification was corrected for the difference in input of RNA by taking reference gene (GAPDH for PBMC or RPS19 for skin samples) to account. For dogs with AD group, evaluation of 2^−ΔΔC^_T_ indicates the fold change in mRNA expressions relative to healthy dogs (healthy dogs = 1). The results were analysed in comparison with the C_T_ (minimum threshold of amplification) value of the target gene and the reference gene (RPS19 or GAPDH as descried earlier). Difference in values was considered statistically significant at *P* < 0.05.

### Statistical analysis

The unpaired *t* test was used to determine the statistical difference between the two groups on GraphPad Prism 8 (San Diego, CA, USA). To establish the degree of relationship between the barrier proteins and cytokines, Pearson’s *r*-correlation statistic was used. Moreover, Pearson’s *r*-correlation statistic was also used to established the degree of relationship between clinical scores of the atopic dogs (CADLI and pVAS) and studied genes expression of barrier proteins and cytokines. The level of statistical significance for all the comparisons made was established at *P* < 0.05. Normality of data distribution was tested using the Shapiro–Wilk test. The data were found to be distributed normally. All data were expressed as mean ± SD.

### Ethics approval

The study was undertaken in compliance of the ethical standards and the study and experimental protocols were approved by the Institutional Animal Ethics Committee of Uttar Pradesh Pandit Deen Dayal Upadhyaya Pashu-Chikitsa Vigyan Vishwavidyalaya Evam Go Anusandhan Sanstahan (DUVASU), Mathura, India (Approval No: IAEC/18/28). Moreover, the authors complied with the ARRIVE guidelines.

### Consent to participate

Samples were collected from client-owned dogs with the written informed consent of the pet owners.

## Results

The CADLI and pVAS scores (Mean ± SD) of atopic dogs were calculated to be 29.55 ± 9.10 and 8.36 ± 1.36, respectively. The mRNA fold expression of skin barrier proteins (*FLG* and *IVL*) is depicted in Table 2. The dogs with AD revealed significantly higher (*P* ≤ 0.01) expression of *FLG* gene as compared to healthy controls. Whereas, significantly (*P* ≤ 0.05) lower expression of the *IVL* gene was revealed by the dogs with AD as compared to healthy controls. Compared with the healthy controls, significantly expression of TNF-α (*P* ≤ 0.01), IL-31 (*P* ≤ 0.05) and IL-13 (*P* ≤ 0.05) genes was quantified in the PBMCs of dogs with AD (Table 3). Out of the 11 atopic dogs included in the present study, expression of the IL-13 gene could be detected only in six dogs, while expression of *IVL* gene was detected in 10 dogs. A non-significant negative correlation of IL-31 and IL-13 with *FLG* expression was observed in dogs with AD. While, a non-significant positive correlation between TNF-α and *FLG* expression was detected. Moreover, a non-significant positive correlation of IL-31, IL-13 and TNF-α with *IVL* was observed in these dogs. The degree of relationship between clinical scores of the severity and the studied genes expression revealed that there was no significant corelation between CADLI, and studied barrier proteins and cytokines expressions. But there was significantly negative correlation between pVAS, and TNF-α (*P* ≤ 0.01) and IL-13 (*P* ≤ 0.05) genes expression. However, significant correlation between pVAS, and studied barrier proteins and IL-31 genes expression could not be established.

**Table 2:** Relative mRNA expression of *Filaggrin* and *Involucrin* in the lesional skin of dogs with AD and healthy controls.

Genes	Change in mRNA expression (Fold change)		*P* values
	Healthy dogs	Dogs with AD	
Filaggrin	1.00	2.07 ± 0.66^a^	0.01
Involucrin	1.00	0.654 ± 0.26^b^	0.05

Data presented are Mean ± SD.

^a^Significantly higher when compared with healthy control.

^b^Significantly lower when compared with healthy control.

**Table 3:** Relative mRNA expressions of TNF-α, IL-31 and IL-13 in PBMCs of dogs with AD and healthy controls.

Genes	Change in mRNA expression (Fold change)		*P* values
	Healthy dogs	Dogs with AD	
Tumour necrosis factor-α	1.00	8.524 ± 4.21^a^	0.011
Interleukin-31	1.00	3.742 ± 1.84^a^	0.028
Interleukin-13	1.00	4.690 ± 2.46^a^	0.041

Data presented are Mean ± SD.

^a^Significantly higher when compared with healthy control.

## Discussion

Filaggrin plays crucial role in maintaining the structural integrity and functions of stratum corneum. *Involucrin* (*IVL*) and loricrin are the CE proteins which help in maintaining the homeostasis of stratum corneum^17^. Filaggrin is the major epidermal protein that has been shown to be an important player in the pathogenesis of AD, and is also known as a modifier of AD^9^. A markedly diminished expression of epidermal differentiation-associated molecules, such as *FLG*, *IVL* and loricrin, has been demonstrated in human patients with AD^27,28^. In human AD patients, an overexpression of type 2 cytokines, especially IL-4 and IL-13, down regulating epidermal barrier proteins has been demonstrated^19,29^. But the mechanism that modulates the expression of *FLG* and integrity of other epidermal barriers in dogs with AD is not well elucidated.

The results of the present study indicated significant increase in mRNA expression of *FLG* and decrease in mRNA expression of *IVL* protein in the lesional skin of dogs with AD. The elevated expression of *FLG* in atopic dogs might be the outcome of compensatory mechanism for restoration of the stratum corneum against the damaging inflammatory cytokines. The expression of *FLG* is up-regulated in AD patients who are wild type for *FLG*. ProFLG is up-regulated in the stratum granulosum. In contrast, *FLG* is reduced in the stratum corneum. This can be explained by reduced activity of proteolytic enzymes that cleave proFLG^30–32^. Significantly decreased expression of *IVL* as observed in the present study could be due to downregulatory action of the elevated type-2 cytokines expression in atopic dogs. In agreement to our study, Santoro et al.^33^ also demonstrated increased *FLG* mRNA expression in experimentally-induced atopic dermatitis in beagle dogs compared to the healthy dogs. In a similar kind of study conducted by Theerawatanasirikul et al.^25^ in atopic dogs, an increased level of *FLG* was observed, however in contrast to our findings, they observed elevated *IVL* expression*.* On the contrary, Roque et al.^10^ performed real time-PCR for quantification of canine *FLG* in the skin of atopic and non-atopic dogs and they reported significant decrease in overall *FLG* mRNA expression in the skin of dogs with AD. Findings of our study are also contrary from the reports in human AD, where decrease in both *FLG* and *IVL* mRNA expressions in the skin lesions has been reported^34–36^. Significant increase in IVL mRNA expression has also been reported in the lesional skin of humans with AD^37^. In an experimental study, non-atopic beagles were sensitized with *Dermatophagoides farinae* (Df) extract but no significant change in *FLG* mRNA expression was observed in skin biopsy^38^. The contradictory findings between our study from those observed in others could be due to species difference or/and variation in the clinical stage of the diseased animals.

To have better insight into the pathogenesis of canine AD, the expression of inflammatory cytokines was undertaken in PBMCs of the atopic and healthy dogs. The mRNA expression of the Th2 cytokines genes, IL-31 and IL-13, and Th1 cytokine gene, TNF-α was quantified using qRT PCR. Markedly higher expression of the IL-13 gene was observed in atopic dogs compared to the healthy ones. In AD dogs, significant negative correlation between the pVAS and IL-13 gene expression was observed which suggests possibly significant role of IL-13 in pathogenesis of canine AD. The result of our study is in agreement with the similar kind of study conducted by Schlotter et al.^39^ in which significantly higher expression of IL-13 mRNA was demonstrated. Similarly, Marsella et al.^40^ also reported significantly higher expression of IL-13 in the lesional skin of dogs with AD. Zheng et al.^41^ used transgenic mouse model which showed the possible direct tissue effects of IL-13 as these mice showed a skin phenotype which reflects AD lesions similar to those observed in humans in several aspects. Hamid et al.^42^ in a study also demonstrated an increased expression of IL-13 mRNA when compared with the skin biopsy specimens from normal control human subject. In the present study, out of 11 atopic dogs participated; the expression of IL-13 genes could only be detected in six atopic dogs. This could be due to the difference in the duration of the clinical disease (chronicity of the disease) in the enrolled atopic dogs before presentation for the clinical investigation.

In the present study, we observed significantly higher expression of IL-31 gene in PBMCs of dogs with AD; which suggests and substantiates the possible role of this cytokine in the pathogenesis of canine AD. An over expression IL-31 in transgenic mice has been attributed to initiation of various hallmark signs of atopic dermatitis for instance increased inflammatory cells infiltration into the cutaneous tissue, marked pruritus, alopecia and cutaneous lesions. Remarkable elevation in IL-31 level in humans with pruritic skin lesions, and positive correlation of IL-31 with the severity of AD in human patients has been documented^43^. Recently, a positive correlation of circulating IL-31 level with the severity of canine AD has also been demonstrated^44^. We observed negative correlation, although non-significant, of IL-31 and IL-13 with *FLG* expression in AD dogs which indicates key role of these Th2 cytokines in the pathogenesis of AD in dogs. Generation of canine IL-31 protein and its biological function in canine systems, as upon administration of the canine IL-31 to dogs, and significant increase in pruritic behaviour has been reported^16^. Therefore, IL-31 seems to play crucial role in inducing pruritus across the species and may be dysregulated in dogs with AD^16^. Results of the present study demonstrated potential association of increased mRNA expression of IL-31 gene with canine AD and is in agreement with the observations in certain recent scientific studies documenting association of IL-31 in dogs with AD^16,44^. Role of IL-31 in human AD has been established at the protein level coupled with the elevated serum IL-31 level in human AD patients^45^. In another study, IL-31 mRNA was detected in a variety of tissues in canines, but not in the skin of dogs with AD and based on these observations, they hypothesized that evaluation of IL-31 protein could be more informative than only looking at the mRNA levels^46^. Altered mRNA expression does not always result in expression at protein level^46^. Our observation of the elevated IL-31 mRNA expression in PBMCs of dogs with AD is in contrast with the aforesaid hypothesis of Mizuno et al.^46^. Activation of inflammatory and itch pathways in dogs with AD and significant up-regulation of genes encoding for Th2 (e.g., IL-4, IL-5, IL-13, IL-31 and IL-33), Th9 (IL-9) and Th22 (IL-22) cytokines in acute canine AD skin lesions has been reported^15^ and their observation is also in agreement with our findings. Significantly higher expression of TNF-α was observed in the present study but there was significantly negative correlation between pVAS and TNF-α genes expression. Maeda et al.^47^ also observed significantly higher TNF-α in the lesional skin than in the non-lesional skin of the dogs with AD. Nuttall et al.^48^ also investigated the mRNA expression of Th1 (TNF-α) from the skin biopsy and detected an increased transcription of TNF-α in lesion atopic skin of dogs. Hence, results of the present study indicate that the studied cytokines- IL-13, IL-31 and TNF-α, have key role in the pathogenesis of canine AD and expression of IL-13 and TNF-α genes in atopic dogs might be down-regulated with increased severity of pruritus.

Based on our findings it may be inferred that dogs with atopic dermatitis manifest skin lesions along with the inflammatory response, and the skin barrier proteins (*Filaggrin* and *Involucrin*) and inflammatory cytokines (TNF-α, IL-31, IL-13) seem to be key players in the pathogenesis of AD in dogs. Thus, the skin barrier proteins and inflammatory cytokines can be considered as the potential targets in therapeutic management of canine AD, however, further studies are warranted at protein transcriptional level.

## Data Availability

Not applicable.
